# The phylogenomic position of the Winghead Shark *Eusphyra blochii* (Carcharhiniformes, Sphyrnidae) inferred from the mitochondrial genome

**DOI:** 10.1080/23802359.2016.1172049

**Published:** 2016-06-20

**Authors:** Pierre Feutry, Peter M. Kyne, Xiao Chen

**Affiliations:** a CSIRO Oceans and Atmosphere, Castray Esplanade, Hobart, Tasmania, Australia;; b Research Institute for the Environment and Livelihoods, Charles Darwin University, Darwin, Northern Territory, Australia;; c Guangxi Key Lab for Mangrove Conservation and Utilization, Guangxi Mangrove Research Center, Beihai, PR China

**Keywords:** *Eusphyra blochii*, mitochondrial genome, Sphyrnidae, threatened species

## Abstract

The complete mitogenome of the Winghead Shark *Eusphyra blochii* (Carcharhiniformes: Sphyrnidae) is determined in this study, which is 16,727 bp with a nucleotide base composition: 31.6% A, 25.7% C, 13.0% G and 29.7% T, containing 37 genes with the typical gene arrangement pattern and translate orientation in vertebrates. Two start codons (ATG and GTG) and two stop codons (TAG and TAA/T) are found in the protein-coding genes. The 22 tRNA genes range from 67 bp (tRNA-*Cys* and tRNA-*Ser*2) to 75 bp (tRNA-*Leu*1). The phylogenetic position showed that *E. blochii* clustered with the *Sphyrna* clade with strong posterior probability (100%).

The hammerhead sharks (family Sphyrnidae) are a small but a highly distinctive group of tropical to temperate coastal and pelagic sharks. The family faces an elevated risk of extinction with several species assessed as threatened on the IUCN Red List of Threatened Species (IUCN 2015). There is also evidence for cryptic species in the Atlantic Ocean (Abercrombie et al. 2005), which presents issues for the identification and monitoring of catches. Molecular identification of hammerhead products can assist catch identification (Abercrombie et al. 2005; Chapman et al. 2009) and is becoming increasingly important as conservation and management measures are implemented for these species, such as the 2013 listing of the three larger-bodied species on the Convention on International Trade in Endangered Species (CITES 2013).

The Winghead Shark *Eusphyra blochii* (Cuvier 1816) occurs in the tropical Indo-West Pacific on continental and insular shelves (Last & Stevens 2009). *Eusphyra blochii* is the most divergent species in the family Sphyrnidae, although its phylogenetic position may vary depending on which gene is analyzed (Lim et al. 2010). Here, we provide the whole mitochondrial genome of *E. blochii* and use it to infer the phylogenomic position of the species.

A tissue sample (fin clip) was collected from a specimen of *E. blochii* captured and released in the lower reaches of the Adelaide River, Northern Territory, Australia, on 07 July 2013. The specimen was a female measuring 70 cm total length. The tissue sample was stored at the CSIRO, Castray Esplanade, Hobart, with voucher no. EBL001. The experimental protocol, data analysis and Bayesian phylogenetic reconstruction follow Chen et al. (2015).

The complete mitochondrial genome of *E. blochii* (Genbank accession no. KU892590) is 16,727 bp with a nucleotide composition (31.6% A, 25.7% C, 13.0% G and 29.7% T), containing typical 37 genes as most vertebrates. There are a total of 23 bp short intergenic spaces and 34 bp overlaps located in gene junctions. Both 12S rRNA (955 bp) and 16S rRNA (1672 bp) are between the *tRNA-Phe* and tRNA-Leu1 genes, separated by the *tRNA-Val* gene. Twenty-two tRNA genes range from 67 bp (*tRNA-Cys* and *tRNA-Ser2*) to 75 bp (*tRNA-Leu1*). Among 30 protein-coding genes, one gene (*CO1*) uses GTG as its initial codon and the remaining genes used the standard ATG codon. Except for four genes (*ND2, ND3, ND6* and *Cytb*) use TAG, the others use TAA/T as terminal codon. The control region is 1088 bp in length, rich in A + T content (66.9%) and poor in G content (13.0%).

Most nodes in the phylogenetic tree were well supported (Figure 1). The basal division was between Scyliorhinidae and the remaining five families. Hemigaleidae and Sphyrnidae were inserted between *Galeocerdo cuvier* (Péron & Lesueur 1822) and other species of Carcharhinidae, which supports an earlier hypothesis by Iglésias et al. (2005) that Carcharhinidae is polyphyletic. The three sphyrnid species formed a well-supported monophyletic clade (Posterior Probability =100%) with *E. blochii* found as sister species to the genus *Sphyrna*. More mitogenomes from other sphyrnid species are needed to fully resolve the phylogeny of this family.

**Figure 1.**
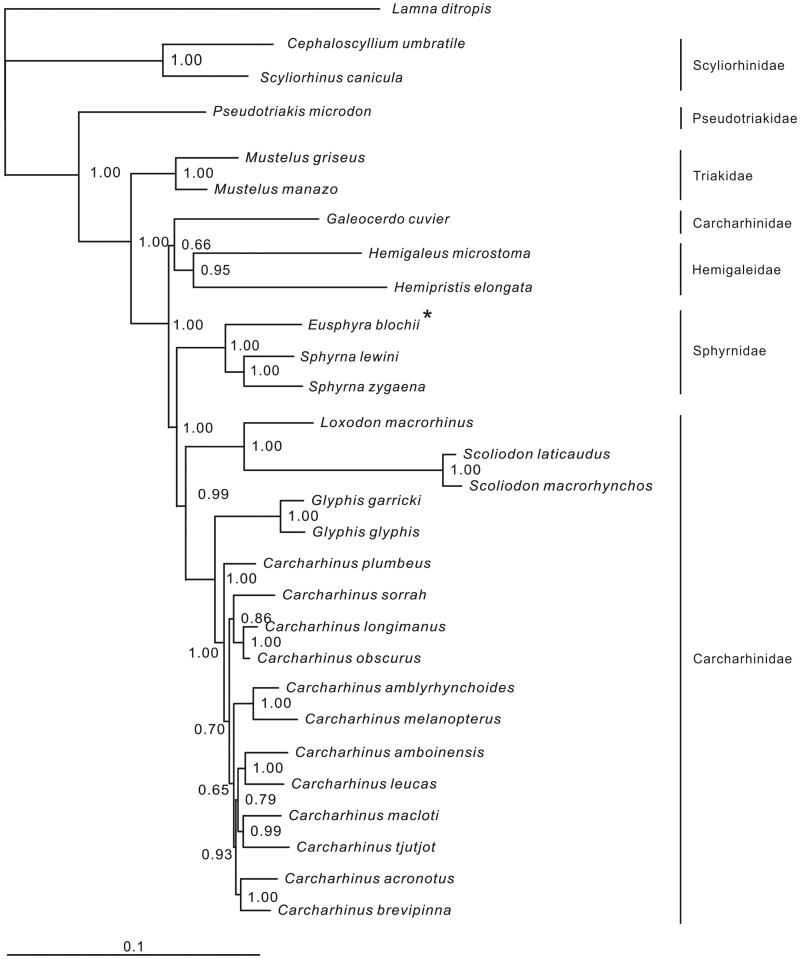
Phylogenetic position of *Eusphyra blochii* (denoted by *). *Lamna ditropis* (KF962053.1) was selected as the outgroup. The 28 species from the order Carcharhiniformes were *Carcharhinus acronotus* (NC_024055.1), *C. amblyrhynchoides* (NC_023948.1), *C. amboinensis* (NC_026696.1), *C. brevipinna* (KM244770.1), *C. leucas* (KF646785.1), *C. longimanus* (NC_025520.1), *C. macloti* (NC_024862.1), *C. melanopterus* (NC_024284.1), *C. obscurus* (NC_020611.1), *C. plumbeus* (NC_024596.1), *C. sorrah* (NC_023521.1), *C. tjutjot* (KP091436.1) *Galeocerdo cuvier* (NC_022193.1), *Loxodon macrorhinus* (KT347599), *Scoliodon laticaudus* (KP336547.1)*, S. macrorhynchos* (NC_018052.1), *Glyphis glyphis* (NC_021768.2), *G. garricki* (KF646786.1), *Mustelus griseus* (NC_023527.1), *M. manazo* (NC_000890.1), *Cephaloscyllium umbratile* (KT003686), *Hemigaleus microstoma* (KT003687), *Hemipristis elongata* (KU508621), *Scyliorhinus canicula* (NC_001950.1), *Pseudotriakis microdon* (NC_022735.1), *Eusphyra blochii* (KU892590), *Sphyrna lewini* (NC_022679.1), *Sphyrna zygaena* (NC_025778.1).
